# Transcriptome Analysis of Potato Leaves Expressing the *Trehalose-6-Phosphate Synthase 1* Gene of Yeast

**DOI:** 10.1371/journal.pone.0023466

**Published:** 2011-08-16

**Authors:** Mihály Kondrák, Ferenc Marincs, Balázs Kalapos, Zsófia Juhász, Zsófia Bánfalvi

**Affiliations:** 1 Agricultural Biotechnology Center, Gödöllő, Hungary; 2 Institute of Genetics and Biotechnology, Szent István University, Gödöllő, Hungary; Purdue University, United States of America

## Abstract

Transgenic lines of the potato cultivar White Lady expressing the trehalose-6-phosphate synthase (*TPS1*) gene of yeast exhibit improved drought tolerance, but grow slower and have a lower carbon fixation rate and stomatal density than the wild-type. To understand the molecular basis of this phenomenon, we have compared the transcriptomes of wild-type and *TPS1-*transgenic plants using the POCI microarray containing 42,034 potato unigene probes. We show that 74 and 25 genes were up-, and down-regulated, respectively, in the mature source leaves of *TPS1-*transgenic plants when compared with the wild-type. The differentially regulated genes were assigned into 16 functional groups. All of the seven genes, which were assigned into carbon fixation and metabolism group, were up-regulated, while about 42% of the assigned genes are involved in transcriptional and post-transcriptional regulation. Expression of genes encoding a 14-3-3 regulatory protein, and four transcription factors were down-regulated in the *TPS1-*transgenic leaves. To verify the microarray results, we used RNA gel blot analysis to examine the expression of eight genes and found that the RNA gel blot and microarray data correlated in each case. Using the putative *Arabidopsis* orthologs of the assigned potato sequences we have identified putative transcription binding sites in the promoter region of the differentially regulated genes, and putative protein-protein interactions involving some of the up- and down-regulated genes. We have also demonstrated that starch content is lower, while malate, inositol and maltose contents are higher in the *TPS1-*transgenic than in the wild-type leaves. Our results suggest that a complex regulatory network, involving transcription factors and other regulatory proteins, underpins the phenotypic alterations we have observed previously in potato when expressing the *TPS1* gene of yeast.

## Introduction

One major approach to improve drought tolerance in crop species is to express genes encoding either metabolic enzymes or transcription factors, which exert their effects through various mechanisms of action [1]. Genes of different origins involved in trehalose metabolism have been used in a number of plant species to improve their drought tolerance [2]. Trehalose, a non-reducing disaccharide consisting of two glucose molecules is a very abundant sugar in nature. In bacteria, yeast and desiccation-tolerant plants it accumulates under osmotic/dehydration stress [3], and helps cells to survive by protecting membranes and proteins [4]. In other plants, however, trehalose is synthesised at an almost undetectable level. In *Escherichia coli*, yeast and plants, trehalose is synthesised in a two-step process. First, trehalose-6-phosphate (T6P) is synthesised from glucose-6-phosphate (G6P) and UDP-glucose (UDPG) by trehalose phosphate synthase (TPS) and then T6P is converted into trehalose by trehalose phosphatase (TPP). In the yeast *Saccharomyces cerevisiae*, an enzyme complex, consisting of trehalose-6-phosphate synthase (TPS) and trehalose-6-phosphate phosphatase (TPP) encoded by the genes *TPS1* and *TPS2*, respectively, is involved in the synthesis of trehalose, while in *Escherichia coli* the corresponding genes are *otsA* and *otsB*. The trehalose biosynthetic genes in plants have been mainly studied in *Arabidopsis*, and three classes of proteins have been distinguished based on domain structure, similarity to the yeast *TPS1* and *TPS2* genes, and the absence/presence of phosphatase boxes in the TPP domain. Ectopic expression of *TPS1* and *otsA* in different plant species and overexpression of *AtTPS1* in *Arabidopsis* improved drought tolerance, but had diverse effects on plant development and resulted in other phenotypic changes in certain species [2], [3].

To improve drought tolerance of potato (*Solanum tuberosum*), we have previously introduced the *TPS1* gene of yeast into the cultivar White Lady, under the control of a drought-inducible potato promoter, *StDS2* [5]. Although the transgenic plants became drought tolerant, it was determined that the transgene was expressed at a very low level even under optimal growth conditions and the transgenic plants displayed certain morphological and physiological changes when compared with the wild-type. For example, they grew slower, had a lower CO_2_ fixation rate and stomatal density was reduced by about 35% [6].

Our observations, and the results of others, highlight the importance of analysis of transgenic plants in order to understand how and why the inserted genes can have such pleiotropic effects. To study this, we have analysed the transcriptome of the wild-type and *TPS1*-transgenic potato plants under unstressed conditions using a microarray, which contains 42K potato unigene probe sequences [7]. Statistical analysis revealed that 99 genes are expressed differentially, and functional annotation revealed that a number of genes are associated with carbohydrate metabolism, while a large proportion (about 42%) of the genes with known function are involved in transcriptional and translational regulation of gene expression. Our results suggest that complex regulation operating at different levels might underpin the observed phenotypical and biochemical changes of the *TPS1*-transgenic potato plants.

## Results

### Physiological changes in potato plants expressing the *TPS1* gene of yeast

Control (*S. tuberosum* cv. White Lady) and two *TPS1-*transgenic lines [6] were grown under well-watered conditions as described in the Materials and methods section. Five physical and biochemical parameters of the lines were then measured, and the results are shown in Table 1. Water and protein content of the transgenic plants did not change compared to the wild-type. Chlorophyll content of the transgenic leaves was slightly, but not significantly, higher than in the wild-type leaves. In contrast, shoot mass and leaf area of the *TPS1*-transgenic lines were, on average, about 35 and 24% lower, respectively, than in the wild-type.

**Table 1:** Measured parameters, as indicated, of potato plants grown under optimal conditions.

Parameters	Wild type	T1	T2
Green mass	59.7±8.8	36.6±6.5^*^	41.7±6.6^*^
Leaf area	1190±155	932±187^*^	827±147^*^
Water content	91.1±0.9	91.5±1.0	90.5±1.1
Chlorophyll content	1.09±0.2	1.20±0.3	1.30±0.2
Protein content	7.95±1.3	8.93±1.5	8.06±1.5

Units for the parameters are: green mass, grams; leaf area, cm^2^; water content, % of the fresh weight; chlorophyll and protein content, mg g^−1^ fresh weight. Samples were collected from three consecutive plant tests. Each biological replicate consisted of three plants. Statistically significant differences from the wild-type were determined using *t* test (*P*≤0.01) and are labelled by asterisks.

### Transcriptome analysis of the *TPS1*-transgenic plants

Previously published observations [6] and the morphological and physiological changes detected in *TPS1*-transgenic plants grown under well-watered conditions described above prompted us to investigate this phenomenon further. To do this, we performed a transcriptome analysis using a potato microarray [8], with which we could monitor expression of a large number of genes simultaneously. Total RNA was isolated from fully expanded leaves of six-week-old plants with the characteristics shown in Table 1. Total RNA was transcribed into fluorescently-labelled cDNA, which was then hybridised to the microarrays in three technical repeats per biological replicates. Images of the hybridised microarrays were analysed by ArrayPro software, and within-array. Loess-normalised data were collected from nine parallel microarrays. After removing bad-spot data, datasets were quantile-normalised between arrays, which adjusts variations in microarray data arising from technical rather than any biological differences (Figure S1). Thus data from the technical and biological replicates become more comparable, a prerequisite for statistical analysis. Between-array-normalised data were then transformed into log2 values and exported into the web-tool ArrayMiner for statistical analysis. Using the empirical Bayesian option of ArrayMiner, 99 genes with a q-value lower than 0.05 were returned, and we consider this to represent significant differences in gene expression between *TPS1*-transgenic and wild-type leaves across all nine arrays. Of these genes, 74 were up- and 25 were down-regulated in the *TPS1*-transgenic plants.

### Annotation of the differentially expressed genes

The 99 differentially expressed genes, which we have identified in the microarray experiments, were exported into the MapMan software for functional annotation. Of these 99 genes, 53 were assigned into different functional groups (bins), while the bin of “not assigned” genes contains 46 genes (Table S1) of which 36 encode unknown, hypothetical or putative proteins. To confirm the annotations of the differentially expressed genes, we performed a BLAST analysis of the potato unigene sequences from which the microarray oligonucleotide probes were designed [7] against the recently completed genome sequence of the doubled monoploid *Solanum phureja* DM1-3 516R44 [Potato Genome Sequencing Consortium, http://www.potatogenome.net/index.php/Main_Page], which is phylogenetically the same species as *S. tuberosum* [8]. Homologies were displayed in the potato genome browser [http://www.potatogenome.net/index.php/Main_Page] and both UniProt [www.uniprot.org] *Solanaceae* entries and corresponding *Arabidopsis* genes of the genomic loci were recorded (Table 2 and Table S2). After this second approach, seven of the MapMan-assigned 53 genes were discarded because of discrepancies between the MapMan and genome data. Thus in total, 46 assigned genes have been obtained (Table 2), and were used in further analyses.

**Table 2:** Functional annotation of differentially expressed genes.

Functional group	MapMan bin code	TPS/wt ratio Log2 value	AT number^a^	Description (short name^b^)
**Photosynthesis**	1.1.1.2	1.32	AT1G67740	Photosystem II core complex proteins (psbY)
	1.3.2	1.69	*AT5G38410*	Rubisco small subunit (RBCS-3B)
	1.3.3	1.89	AT1G56190	Phosphoglycerate kinase (PGK1)
	1.3.6	1.60	*AT4G38970*	Fructose-bisphosphate aldolase (ALDP1)
**CHO metabolism**	2.1.2.5	1.89	**AT3G08580**	Adenine nucleotide carrier protein (ANT1)
	2.2.1.5	4.64	AT4G02280	Sucrose synthase (SUS3)
**TCA / org**	8.1.1.1	2.47	AT1G01090	Pyruvate dehydrogenase E1 alpha subunit (PDH-E1)
**Hormone metabolism**	17.5.3	3.47	**AT3G16050**	A37 protein, pyridoxine biosynthesis protein (PDX1.2)
	17.6.3	4.32	*AT1G75750*	Snakin2 (SN)
**Stress**	20.2.1	−2.57	AT1G59860	17.6 kDa class I heat shock protein (HSP17.6A-CI)
**Redox**	21.5	−1.87	*AT1G17020*	Leucoanthocyanidin dioxygenase (ANT17)
	21.5	1.12	AT5G06290	Thioredoxin peroxidase (TPX1)
	21.6	1.69	*AT4G35090*	Catalase (CAT2)
**Nucleotide metabolism**	23.4.10	2.39	**AT4G09320**	Nucleoside diphosphate kinase 1 (NDPK I)
**Miscellaneous**	26.1	2.64	**AT1G15390**	Peptide deformylase (PDF1A)
	26.7	2.64	AT5G16990	Allyl-alcohol dehydrogenase (ADH)
	26.8	2.39	AT5G22300	Bifunctional nitrilase/nitrile hydratase (NIT4B)
	26.21	2.83	AT2G44300	Non-specific lipid transfer protein
	26.21	2.47	*AT2G10940*	Proline-rich protein
	26.24	2.39	AT2G32030	GCN5-related N-acetyltransferase (GNAT)
**RNA**	27.1.19	2.00	**AT3G44260**	CCR4-associated factor
	27.3.2	−1.91	AT1G14510	Alfin-like transcription factor (FIN1)
	27.3.9	−3.81	**AT5G66320**	AG-motif binding protein 4/C2C2 GATA Zinc finger TF (AGP4)
	27.3.24	−5.36	AT5G60910	Agamous-like AGL8 MADS-box protein (POTM 1-1)
	27.3.24	−1.99	AT4G24540	Agamous-like AGL24 MADS-box protein (MADS11)
	27.4	3.64	*AT4G24770*	31-kDa RNA binding protein (28RNP)
	27.4	2.06	AT1G54080	Oligouridylate binding protein
	27.4	−2.89	AT3G15010	Nuclear ribonucleoprotein A1
**DNA**	28.1.3	2.64	AT4G40030	Histone H3.2 (H3)
**Protein**	29.2.2	2.64	AT5G27700	40S ribosomal protein S21 (RPS21e)
	29.2.2	−5.70	*AT5G64140*	40S ribosomal protein S28 (RPS28)
	29.2.3	1.39	**AT4G00820**	Calmodulin binding protein (SUI1B)
	29.2.4	7.63	**AT1G07940**	Calmodulin binding / translation elongation factor
	29.5	1.78	AT5G45390	ATP-dependent Clp protease (CLPP)
	29.5.11.3	2.55	*AT2G02760*	Ubiquitin-protein ligase (UBC2)
	29.5.11.4.2	3.47	**AT3G14250**	Zinc finger (C3HC4-type RING finger) ubiquitin conjugating enzyme
	29.5.11.4.3.2	−8.48	AT1G15670	Kelch repeat-containing F-box family protein
	29.5.11.20	2.06	AT3G27430	Proteasome subunit beta type-7-A (PBB1)
	29.5.11.20	2.32	AT1G47250	Proteasome subunit alfa type (PAF1)
**Signalling**	30.7	−2.18	*AT5G38480*	14-3-3 protein 4 (TFT4)/GRF3-like
**Cell**	31.1	2.64	AT5G56600	Profilin (PRO)
	31.1	2.64	**AT5G09810**	Actin 7 (ACT7)
**Transport**	34.1	3.83	AT4G02620	Vaculoar ATPase subunit F
	34.1.1	1.39	AT1G19910	V-type proton ATPase 16 kDa proteolipid subunit (AVA-P2)
	34.12	2.74	AT1G55910	Putative zinc transporter (ZIP11)
	34.99	1.74	*AT5G65380*	Multidrug resistance pump

a AT numbers in bold and italics indicate common genes which are regulated in the same and the opposite manner, respectively, in mature leaves of *TPS1*-transgenic potato plants (this study) and *otsA*-transgenic *Arabidopsis* seedlings [24]. Underlined numbers label genes with corresponding *S. tuberosum* and/or other *Solanaceae* entries in the UniProt database (Table S2). ^b^Wherever available, either *S. tuberosum* or other *Solanaceae* gene/protein name obtained from the UniProt database is displayed (see also Table S2).

*TPS1*-transgenic potato plants display reduced growth and CO_2_ fixation rate [6], which may be linked to carbohydrate metabolism at the molecular level. Therefore, the seven genes in Table 2, which are associated with carbon fixation and metabolism, and are up-regulated in the *TPS1*-transgenic plants, may be significant. Four of these genes are associated with photosynthesis, two with major carbohydrate metabolism, and one with the tricarboxylic acid cycle. Of the photosynthesis-associated genes, *pbsY* encodes a photosystem II thylakoid membrane protein [9] and *RbcS* is a nuclear gene family member encoding small subunits of the Rubisco complex localised in the chloroplast stroma [10]. Phosphoglycerate kinase (PGK1) and fructose bisphosphate aldolase (ALDP1) are also stromal proteins, both having a function in the Calvin cycle, while ALDP1 is also involved in glycolysis [11]. One of the major carbohydrate-metabolism-associated genes encodes a sucrose synthase, which catalyzes the conversion of sucrose into UDP-glucose and fructose. The particular gene (*SUS3*), which is up-regulated in *TPS1*-transgenic plants, was shown to be expressed at the highest levels in stems and roots of non-transgenic plants [12]. The other gene in this functional group is an adenine nucleotide carrier protein [13].

A large proportion (about 42%) of the assigned genes belongs to functional groups of RNA, DNA and protein-associated genes. One of these is an Alfin1-like PHD-finger transcription factor, a second is an AG-motif binding protein 4 (AGP4), similar to the GATA zinc-finger transcription factor GATA5 and two others are similar to the MADS-box TFs AGL8 and AGL24. All four TF genes were down-regulated in *TPS1*-transgenic plants. In general, PHD-finger proteins are thought to be chromatin mediated transcriptional regulators, but one of them, Alfin1 is a promoter-binding TF [14]. GATA factors are zinc finger domain-containing DNA binding TFs, which are involved in diverse developmental and environmental pathways, including responses to light. It is worth to note that the corresponding *Arabidopsis* protein of AGP4, GATA5, is expressed in all mature plant tissues at an almost constant level, and is up-regulated in light-grown plants [15]. MADS-box proteins are DNA-binding TFs involved in plant developmental processes, including floral development and transition between vegetative and reproductive phases [16]. Based on the UniProt database [http://www.uniprot.org], there are two corresponding proteins of AGL8 in potato, POTM1 and SCM1, which are 96% identical to each other. Suppression lines of *POTM1*, which belongs to the same MADS-clade as *AGL8* [17], produce truncated shoot clusters from stem buds and exhibit enhanced axillary bud growth instead of producing a tuber [18]. The other MADS-box protein, AGL24, is homologous to StMADS11 and belongs to the StMADS11 clade of MIKC-type MADS-box proteins [19]. *STMADS11* was isolated from *S. tuberosum*, and is expressed in all vegetative tissues [17]. In the protein-associated functional group, there are two genes, which are up-regulated in *TPS1*-transgenic plants and encode calmodulin-binding proteins. Calmodulins are Ca^2+^-binding proteins, which interact with a large number of structurally and functionally diverse proteins [20]. Five genes, four of which are up- and one down-regulated, encode proteins involved in the ubiquitin-proteasome pathway. Two of these proteins are a ubiquitine-conjugating (E2) and a ubiquitine-ligase (E3) protein working in a cascade to ubiquitinate target proteins, which then are transferred into the 20S proteolytic unit of the 26S proteasome for degradation [21]. One protein is a Kelch repeat-containing F-box family protein, which is a subunit of the E3 ubiquitine-ligase complex [22]. Two other genes encode 20S proteasome components. E2 and E3 proteins are encoded by large gene-families [23] and different combinations of these proteins provide for very selective ubiquitination and consequently degradation of cellular proteins in proteasomes [21]. A CLP protease (CLPP) was also up-regulated in *TPS1*-transgenic plants. CLPP is one of those chloroplast proteases, which is located in the stroma, and forms a proteolytic complex with other proteases and is assumed to be a housekeeping protease [24].

Another gene in Table 2 encodes for a protein that is homologous to GRF3 of *Arabidopsis*, which is a (ψ)-type 14-3-3 protein (TFT4) expressed in stems, leaves and flowers. 14-3-3 proteins, which are ubiquitous in animals and plants, bind commonly, but not exclusively to phosphorylated target proteins and are considered of great significance because they act as central regulators of metabolism and signalling in plants [25].

### Verification of microarray results

**Figure 1:**
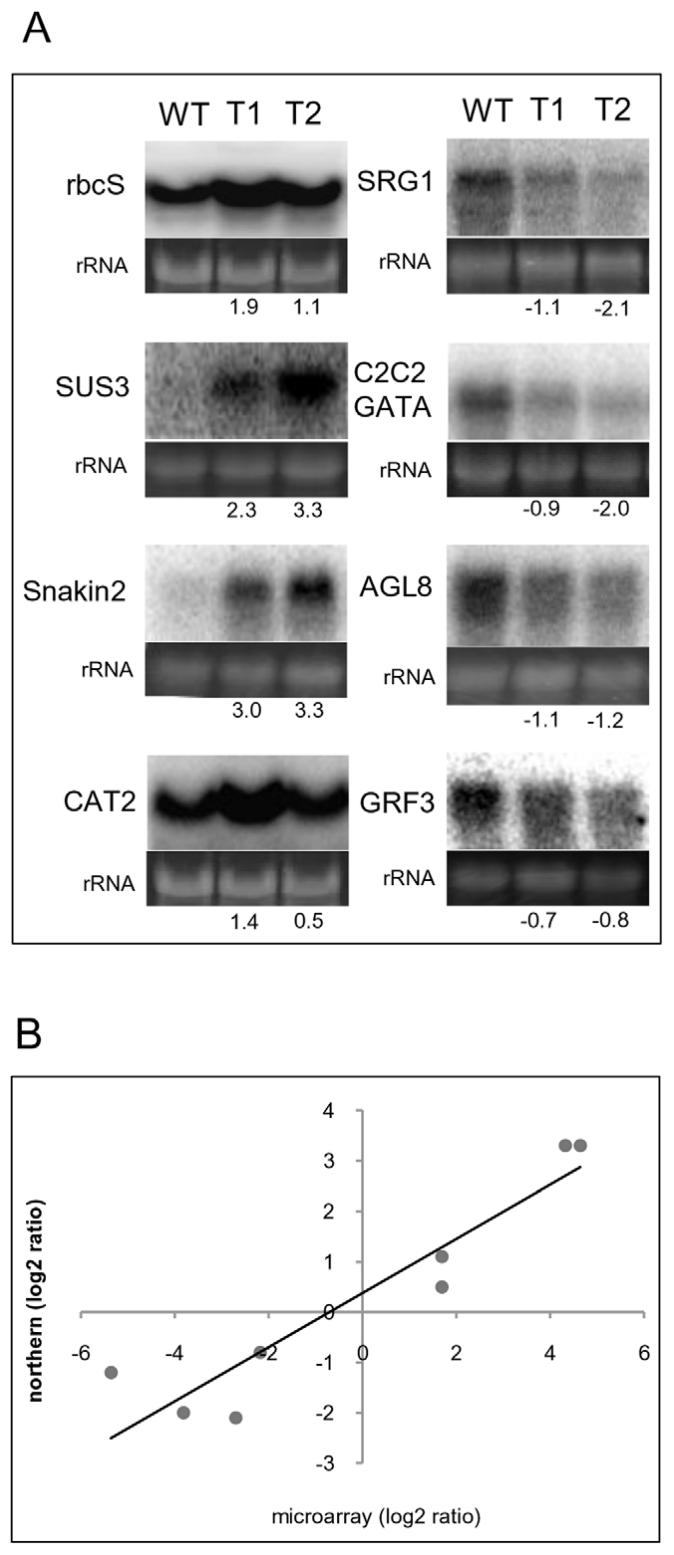
Validation of microarray results. A) RNA gel blot analysis of selected genes. Phosphorimage analysis was used to quantify the intensity of hybridisation. The expression ratios between T1, T2 and wild-type plants are shown below the lanes as log2 values. Ethidium bromide-stained rRNA bands are shown as loading controls. WT, wild-type; T1, T2, two independent *TPS1*-transgenic lines. B) Correlation between microarray and northern results in T2/wild-type comparison. A statistically significant correlation (r = 0.9369, p = 2.376e^−06^) was obtained for all genes tested.

Because the two *TPS1*-transgenic lines did not show any significant differences in terms of the measured physical and biochemical parameters (Table 1), only one of the lines, T2, was used for the microarray experiments to compare its transcriptome with the non-transformed control. However, to check the reliability of our microarray results, both *TPS1*-transgenic lines were analysed in RNA gel blot analysis. For this, total RNA, isolated from the leaves of the wild-type and *TPS1*-transgenic lines, was separated on agarose gels, transferred to membranes and probed with gene-specific radioactive probes for eight of the differentially regulated genes. Thus the expression of about 17% of the assigned genes was assessed by RNA blot analysis. After scanning the autoradiographs, the ratio of the signal between the *TPS1*-transgenic and the wild-type lines was calculated. Line T1, whose transcriptome was not examined by microarray, gave similar results to line T2 (Figure 1A) indicating that, very likely, gene expression in both lines follows the same pattern. Although the expression ratios for all tested genes were slightly different in the microarray and the RNA gel blot experiments, they had a strong positive correlation with an r-value of 0.9369 (Figure 1B). Thus our microarray results can be assumed to be correct and reliable.

### Leaf carbohydrate and starch content

The microarray results showed that a sucrose synthase gene (*SUS3*) and six other genes associated with photosynthesis and carbon metabolism are up-regulated in *TPS1*-transgenic leaves. We investigated, therefore, the relative levels of major carbohydrates and starch in the *TPS1*-transgenic leaves as compared with the wild-type levels.

**Figure 2:**
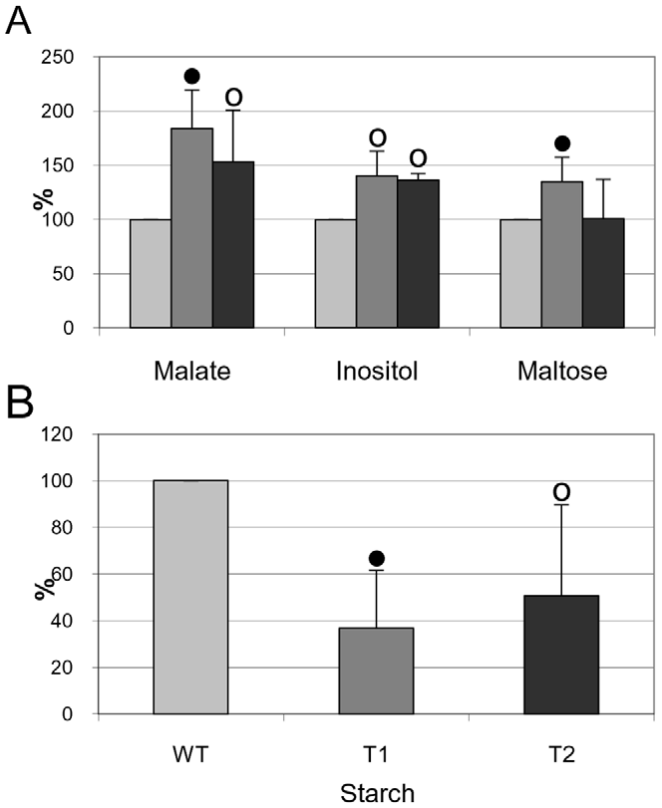
Relative amounts of sugars (A) and starch (B) in wild-type (WT) and *TPS1*-transgenic (T1, T2) leaves. Bars and error bars represent the mean ± SE derived from three independent experiments. Filled and open circles denote differences significant at *P* = 0.01 and *P* = 0.05 (*t* probe) levels, respectively, when compared with the wild-type. The carbohydrate concentrations in wild-type leaves in the three independent experiments were as follows: malate 19.4, 16.7, 17.6 µmol/g FW; inositol 0.89, 0.9, 0.94 µmol/g FW; maltose 0.51, 0.53, 0.52 µmol/g FW; starch 4.4, 1.7, 0.5 µmol hexose equivalent/g FW. These are regarded as 100% values for comparison with the equivalent samples from the transgenic leaves.

Carbohydrates and starch were extracted from the same pool of leaves used for microarray analysis. GC-MS analysis revealed that the amounts of D-fructose, D-galactose, D-glucose, sorbitol, and sucrose are largely similar in each line (data not shown), while the amounts of inositol and maltose, and the organic acid, malate, are increased in the *TPS1*-transgenic lines (Figure 2A). In terms of starch content, the levels in the wild-type showed a large variation between the biological repeats (4.4, 1.7, 0.5 µmol hexose equivalent/g FW), but were always proportionally higher than in *TPS1*-transgenic leaves (Figure 2B). Photosynthetic partitioning into starch is finely regulated, and the amount of carbohydrate stored is dependent upon the environmental conditions experienced by the plant, particularly day length [26]. Thus small differences in day length, light intensity and temperature in the greenhouse during the three consecutive plant tests may explain the variation in starch content. When compared with starch, much less variation was detected in malate (17.9±1.37 µmol/g FW), inositol (0.91±0.03 µmol/g FW) and maltose (0.52±0.01 µmol/g FW) content. Although *TPS1* mRNA was detectable even under unstressed conditions in the transgenic plants [6], no trehalose (<0.006 µmol hexose equivalent/g FW) was detected either in wild-type or *TPS1*-transgenic leaves. Under water-deficit-stress, the level of *TPS1* mRNA slightly increased compared to well-watered conditions [6], but trehalose was still undetectable (data not shown). This may be due to the high trehalase activity detected in dicotyledonous plant species [2].

### Interaction analysis

The large proportion of genes associated with transcriptional and translational regulation identified in the microarray experiments prompted us to investigate some elements of the putative regulatory network that may underpin the observed differential gene expression. Unfortunately, in potato, such interaction information is very limited. For example, protein orthologs from solanaceous species are not listed in the databases of OMA (http://www.cbrg.ethz.ch/research/orthologous/index), Roundup (http://roundup.hms.harvard.edu/site/index.php), and BioGRID (http://thebiogrid.org) for protein-protein interactions. In another interaction database, IntAct (http://www.ebi.ac.uk/intact/main.xhtml), there are only 27 interactions out of 266,855 which have *S. tuberosum* proteins as the interacting partner, but only a fraction of these (seven interactions) are real binary interactions; the others are co-complexes. Thus acknowledging the problems associated with gene orthology in general, and in particular in the case of gene families [27], our analyses had to be based on putative *Arabidopsis* orthologs of the assigned potato sequences (Table 2). We assumed that orthologs have the same function in different species [28], and so for the protein-protein interaction analyses, we chose only those genes (28 in all) which have corresponding genes in both *Arabidopsis* and *Solanaceae* (Table S2), while putative TF-binding site analyses were performed for all genes in Table 2.

Firstly, we have searched the AthaMap database for transcription factors, which bind to the −500 to +50 region of the assigned genes. In particular, we were interested to see whether the four transcription factors (Alfin1, C2C2 GATA, AGL8, AGL24) that have been detected as differentially expressed genes in our microarray experiments bind to the promoter region of putative orthologs of the assigned potato genes. The results of the search are shown in Table S3. Alfin1 is predicted to bind to the promoter region of all but seven genes. Bound promoters include *AGL8*, *AGL24* and *C2C2 GATA*, but not the *Alfin1* gene itself, suggesting that Alfin1 does not regulate its own synthesis at the transcription level. At the time of writing this manuscript, there was no entry in the AthaMap database for the two MADS-box proteins, AGL8 and AGL24, the orthologs of which are down-regulated in the *TPS1*-transgenic plants. However, the MADS-box protein, AGL15, does bind to the promoter region of all four TFs, while another, AGL2 binds to the promoter of the *C2C2 GATA* gene (Table S3).

We also performed an additional search of the AtcisDB database. While AthaMap returns information about TFs which bind to the promoter region of *Arabidopsis* genes, AtcisDB contains information about the DNA motifs to which the TFs bind. This search revealed the presence of CArG MADS protein-binding boxes in the promoter of *AGL8* and *Alfin1*. GATA binding sites were identified in the promoter region of all four TFs. In addition to these specific findings, a number of additional putative TF-binding sites were identified in the promoter region of all assigned genes, revealing a very complex matrix of the assigned genes, the TFs and their binding sites (Table S4).

**Figure 3:**
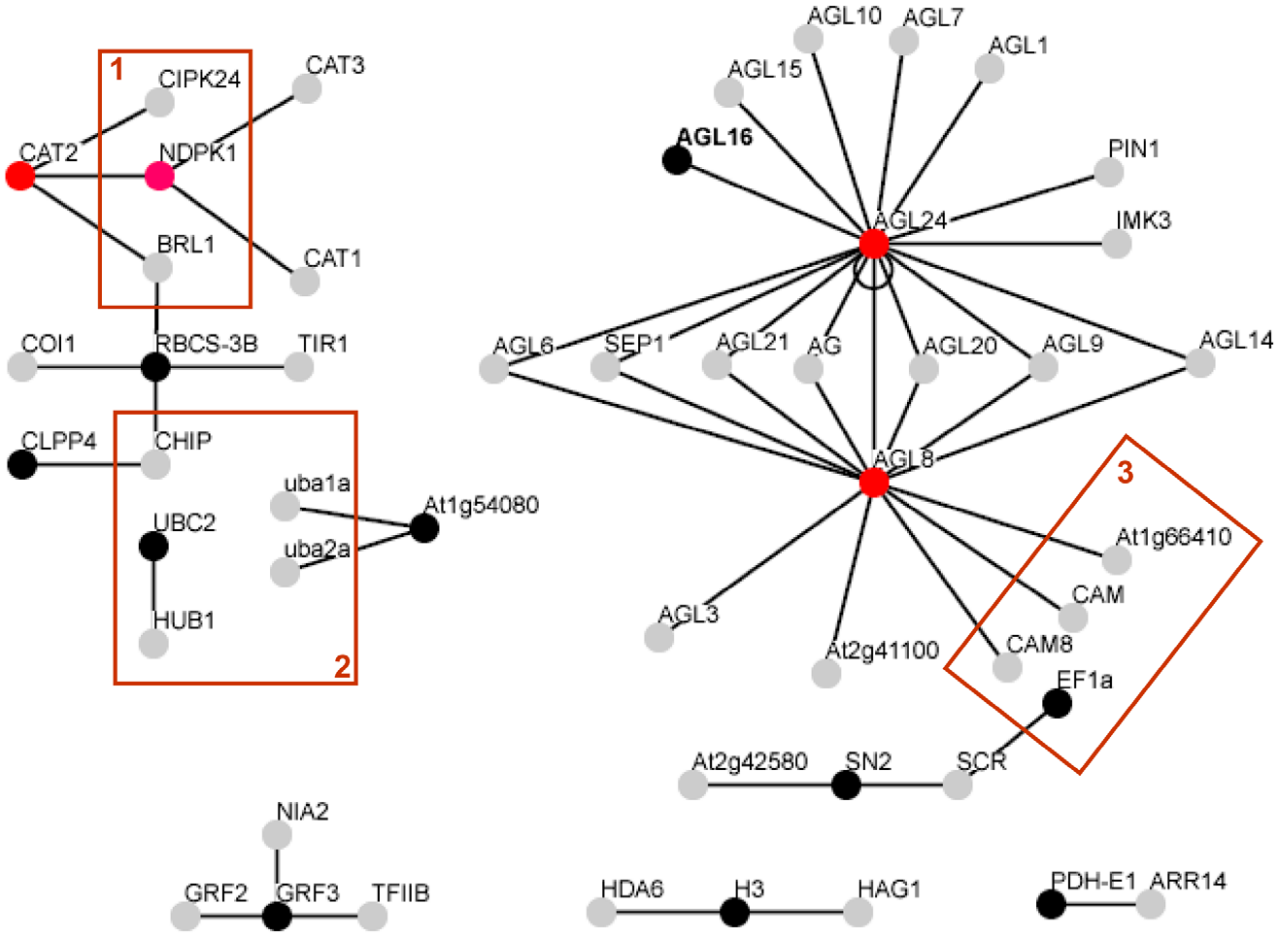
Networks of interacting proteins. Proteins encoded by differentially expressed genes from the microarray experiments and their interacting partners are labelled with black and grey circles, respectively. Proteins which are present on both the microarray and the partner lists (Table S5) are labelled by red circles. Proteins with similar functions are boxed: 1, kinases; 2, ubiquination; 3, calmodulins/calmodulin binding. AGL16, a guard cell-specific transcription factor interacting with AGL24, an Agamous-like transcription factor, is in boldface (see text for explanation). AGL24 interacts with itself too, indicated with a circled line. For protein descriptions and available *Solanaceae* protein names see Table S5.

As the part of our analysis, we have investigated whether any protein-protein interactions occur between the proteins encoded by the up- and down-regulated genes. For this, the BioGRID and IntAct databases were interrogated using the Locus ID of the corresponding *Arabidopsis* genes of 28 potato genes (Table 2). We have found that 13 proteins have proven binary interactions in the databases. All of these interactions are shown in Figure 3 and in Table S5. We have identified two interactions in which both partners are proteins encoded by orthologs of differentially expressed potato genes. Namely, these are the NDPK1/CAT2, and the AGL8/AGL24 interactions. The biggest network of interacting proteins is associated with the MADS-box proteins, AGL8 and AGL24, which also interact with each other. They have twelve and 14 interacting partners, respectively, with seven of these proteins common to both. Their interacting partners are mostly Agamous, Agamous-like or other MADS-box proteins, but AGL8 also interacts with three calmodulins. MADS-box proteins form homo- or hetero-dimers and are considered as combinatorial transcription factors [29], which explains the interaction of AGL8 and AGL24 with a number of other MADS-box proteins. The second largest protein network consists of eleven proteins involved in carbohydrate and nucleotide metabolism, redox and protein processes. Some proteins of this network are connected by some common interacting partners, such as a protein kinase and a ubiquitin-protein ligase. The 14-3-3 protein has three interacting partners, a nitrate reductase, another general regulatory factor (GRF2) and a transcription initiation factor. Some minor interactions, involving 2 or 3 interacting proteins were also identified.

## Discussion

In a previous paper we have reported that introducing the *TPS1* gene of yeast into *S. tuberosum* cv. White Lady resulted in drought-tolerance accompanied by certain pleiotropic effects, which could be observed even under well-watered conditions [6]. In the *Solanaceae*, similar studies have been undertaken with tobacco and tomato by introducing either the same *TPS1* gene or its *E. coli* ortholog, *otsA*, under the control of a constitutive (CaMV35S), tissue specific (Rubisco, patatin, 16SrRNA) or drought-inducible (AtRAB18) promoter [2], [3]. Almost all of the transformed plants displayed drought-tolerance, but the strong constitutive promoter combined with either *TPS1* or *otsA* caused phenotypical changes in all three species, similar to those observed by us. While these previous studies focused on the biochemical aspects of drought-tolerance in transgenic solanaceous plants, in the current study we have applied genomic and bioinformatic approaches to investigate the differences between wild-type and transgenic potato plants and in doing so have identified 99 genes, which are either up- or down-regulated in the leaves of *TPS1*-transgenic potato plants when compared with the wild-type under well-watered conditions.

Previously, it has been shown that T6P inhibits SnRK1 activity in extracts of *Arabidopsis* seedling and leaf tissues of different ages, with the exception of mature leaves [30]. This study also found that seedlings expressing *otsA* show opposite regulation of the SnRK1 target genes. By comparing this study with our microarray results we have found 22 assigned potato genes out of 46 whose expression was changed both in *otsA*-transgenic *Arabidopsis* seedlings and in mature leaves of *TPS1*-transgenic potatoes. However, 10 of these displayed an inverse regulation (Table 2) suggesting that further work is necessary to unequivocally establish the effect of T6P on SnRK1 in mature leaves of potato.

The expression of two ortholog genes, *GRF3* and *TFT4*, which encode a member of the 14-3-3 protein family, is reversed in the *otsA* and *TPS1*-transgenic plants, respectively. *TFT4* is down-regulated in *TPS1*-transgenic plants which also displayed reduced leaf area, while *GRF3* influences leaf growth in *Arabidopsis* [31]. Among the proteins with which binding of 14-3-3 proteins has been demonstrated is a cauliflower TPS [32] while TPS5, 6, and 7 of *Arabidopsis* also bind to 14-3-3 proteins if the Ser22 and Thr49 residues are phosphorylated [33]. Since the TPS1 protein of yeast is 40% identical with TPS5, and TPS1 contains Ser and Thr residues at the same locations as the *Arabidopsis* TPS isoforms, we postulate that phosphorylation of yeast TPS1 and its interaction with 14-3-3s may also exist in potato. Binding of yeast TPS1 to 14-3-3 can influence the activities of house-keeping proteins such as nitrate reductase whose interaction with GRF3 has been demonstrated in *Arabidopsis* (Figure 3). The down-regulation of *TFT4* might result in reduced 14-3-3 availability leading to an imbalance in ion homeostasis and hormone signalling in which 14-3-3s have well understood functions [25].

Four transcription factors, similar to two MADS-box proteins (AGL8 and AGL24), a GATA factor and an Alfin1 TF, are down-regulated in the leaves of *TPS1*-transgenic plants. These four TFs may bind to the promoter regions of other differentially expressed genes in a very complex pattern (Table S3 and S4). In addition, the two MADS-box proteins interact with a number of other MADS factors (Table S5), which is in good agreement with the proposed combinatorial regulation of vegetative development by MADS factors [19].

Two genes, *SUI1B* and *EF1a*, that encode proteins, which bind Ca^2+^-binding calmodulin proteins, are up-regulated in *TPS1*-transgenic potato plants. In addition, AGL8 also interacts with three calmodulins (Figure 3 and Table S5) and together this indicates that Ca-signalling might have an important role in the *TPS1*-transgenic plants. We also propose that protein phosphorylation *via* calmodulins, which has an important role not only in regulatory cascades but also in protein degradation, may be affected. We have also found that several genes, encoding proteins involved in the ubiquitin-proteasome pathway, are differentially expressed, mostly up-regulated, in the *TPS1*-transgenic plants when compared with the wild-type (Table 2). Furthermore, an ubiquitin-ligase (UBC2) and the Rubisco small subunit (RBCS-3B), the genes for which are up-regulated in *TPS1*-transgenic plants, interact with HUB1 and CHIP ubiquitin-ligases, respectively. Altogether there are five proteins in the protein networks which are involved in ubiquitination. In addition to their numerous roles in regulation, 14-3-3 proteins can either inhibit or promote degradation of phosphorylated protein to which they bind [34], and so the down-regulation of a gene encoding such a protein can influence the turnover of a number of proteins. Together, these findings indicate that posttranslational regulation might also have a role in the development of the phenotypes observed in the *TPS1*-transgenic plants.

In terms of regulatory cascades, it is now well established that plants respond to environmental stresses *via* mechanisms involving sugar signalling and hormonal factors [35]. It is thus not surprising that we have found hormone metabolism and stress-related genes that are differentially regulated in the *TPS1*-transgenic plants even under well-watered conditions (Table 2). In addition, a number of genes assigned into other functional groups are known to be stress-responsive (data not shown). A recent example of such interlocking regulatory cascades is the observation that mutant plants with impaired nitrate reductase are also dehydration resistant [36].

It appears to be inconsistent that a Rubisco small subunit gene is up-regulated (Table 2), while CO_2_ assimilation is reduced in the *TPS1*-transgenic plants [6]. However, it has been shown that the protein abundance and activity of Rubisco is not always correlated with changes in the amount of *rbcS* transcript. Moreover, the CO_2_ fixing reaction catalysed by Rubisco is reversible and molecular oxygen can also be a substrate of the Rubisco complex [37]. It is possible therefore, that the up-regulation of *rbcS* has an effect on these reactions and together with the inherently complex regulation of Rubisco results in the net reduction in CO_2_ assimilation, which may also be the primary reason of the decreased starch content of leaves. Another explanation for the observed reduced CO_2_ assimilation might be the lower stomata density observed in *TPS1*-transgenic plants [6]. It has been shown that the density and development of stomatal complexes on the epidermis of *Arabidopsis thaliana* leaves depend, in part, on the microRNA-mediated regulation of *AGL16* [38], which is a member of the MADS-box protein family and expressed in guard cells [39]. In this respect it is intriguing therefore that AGL16 is among the interacting partners of AGL24 whose corresponding gene (*StMADS11*) is down-regulated in *TPS1*-transgenic plants. Assuming that these proteins exist in potato, an altered interaction between them, due to the down-regulation of *StMADS11*, might be an alternative explanation for the lower stomata density and reduced CO_2_ assimilation rate of *TPS1*-transgenic leaves.

Although, the level of pyruvate dehydrogenase, phosphoglycerate kinase and fructose-bisphosphate aldolase mRNAs are increased in *TPS1*-transgenic potato leaves no significant changes in levels of major carbohydrates, glucose, fructose, and sucrose were detected while the amounts of malate, inositol and maltose were increased. Pathways interconnecting these enzymes and metabolites are shown in Figure S2. It is worth noting that carbohydrate metabolism in drought tolerant transgenic plants harbouring different trehalose biosynthetic enzymes can be very different. For example, introducing the *otsA* and *otsB* genes into rice resulted in slightly elevated levels of glucose, fructose and sucrose under both well-watered and drought-stress conditions [40], while in our potato lines transformed with the *TPS1* gene the levels of these sugars remained quite constant under all conditions. This very likely reflects certain differences in the carbohydrate metabolism between dicots and monocots.

So, putting all information together we propose that molecular interactions and complex regulatory mechanisms at transcriptional, translational and post-translational levels underpin the pleiotropic effects in drought-tolerant potato, harbouring the *TPS1* gene of yeast.

## Materials and Methods

### Ethics statement

This work did not raise ethical issues.

### Plant material and growth conditions

*Solanum tuberosum* cv. White Lady wild-type and *TPS1*-transgenic plants T1 and T2 [6] were vegetatively propagated from single-node stem segments in tissue culture and maintained at 24°C under a 16 h light / 8 h dark regime on RM medium [41]. Six-week-old plants were transferred to pots containing A260 sterile soil (Stender, Germany) and were grown in a greenhouse in summer, under natural light, at 20–28°C, and at a soil water content of 70%. After six weeks in the greenhouse, mature source leaves of vegetative growth-phase plants were sampled for further analysis, four hours after sunrise.

### Physical and biochemical measurements

To determine total shoot mass, the entire aerial part of plants were harvested by excising the stem one cm above the soil and the collected material weighted. The moisture content of leaves was calculated from the fresh and dry weight and expressed as the percentage of the fresh weight. To measure leaf area, freshly collected leaves were scanned and their area was determined using PhotoShop software. To measure chlorophyll and protein contents, leaves were powdered in liquid nitrogen and 500 mg of the powder was vortexed in 5 ml of ice-cold acetone, followed by centrifugation at 13000 rpm for 10 min at 4°C to remove cell debris. The supernatant was neutralised with an equal volume of 1 M Tris-HCl, pH 8.0, and then the absorbance was measured at 645 and 663 nm. Chlorophyll content was calculated from the absorbance data and expressed as mg per g fresh weight. For protein measurement, 100 mg of the powdered leaf material was vigorously mixed with 400 µl of 0.1 M Na-phosphate buffer, pH 7.8, followed by centrifugation at 13000 rpm for 30 min at 4°C. The concentration of the total soluble proteins in the supernatant was measured using a dye-binding method [42].

Extraction, derivatisation and analysis of potato leaf carbohydrates were carried out as described by [43] using a quadrupole-type GC-MS system (Finnigan Trace/DSQ, Thermo Electron Corp.). The chromatograms and mass spectra were evaluated using the XCALIBUR software (Thermo Electron Corp.) and the NIST 2.0 library.

Starch was isolated from 150 mg of leaf tissue powdered in liquid nitrogen by incubation in 1 ml of a solvent containing 80% (v/v) ethanol and 5% (v/v) formic acid at 80°C for 10 min. After centrifugation at 13000 rpm for 10 min the supernatant was removed and the pellet resuspended in 1 ml of 80% (v/v) ethanol and incubated at 80°C for 5 min. The pellet was harvested again by centrifugation and after washing twice with 80% (v/v) ethanol, the starch was solubilised with 400 µl of 0.2 N KOH at 95°C for 1 h. The solution was neutralised by 70 µl of 1N acetic acid and cleared by centrifugation after which 100 µl of the supernatant was mixed with 10 µl of Lugol solution (2 g KI, 1 g I_2_ dissolved in 150 ml distilled water) in a 96-well microplate, and the developed colour measured at 595 nm in a Multiskan EX (Labsystems) microplate reader. A calibration curve was prepared, using solubilised corn starch (Sigma) as control, to determine starch content in the leaf samples.

### RNA isolation and cDNA synthesis

For the microarray experiments, plants were grown in three biological replicates, each containing six plants. All fully expanded leaves of all plants of each replicate were pooled and then total RNA was extracted from the pools as described [44]. Fluorescently labelled cDNA was synthesised from 20 µg total RNA using a SuperScript Plus Direct labelling kit (Invitrogen), according to the manufacturer's instructions. Alexa Fluor 647- and 555-labelled dUTPs were used for the transgenic and wild-type samples, respectively, and anchored oligo (dT) was used as the primer. The labelled cDNA was purified using a MinElute PCR purification kit (Qiagen), and was quantified using a NanoDrop spectrophotometer. The cDNA yield varied between 1.06 and 2.31 µg, while specific dye incorporation was between 25 and 113 pmol dye/µg cDNA.

### Microarray processing

POCI potato microarrays (4×44 K; [7]) were purchased from Agilent. One microgram of each of the Alexa Fluor 647- and 555-labelled cDNAs were combined, dried in a SpeedVac, and dissolved in 20 µl of water. The hybridisation mixture was set up using a Gene Expression Hybridisation Kit (Agilent) and applied to the microarray, which was then hybridised at 65°C for 17 h, followed by two subsequent washes in the appropriate Agilent wash solutions for one minute each at RT and 37°C, respectively. The dried microarrays were then scanned with a Genetix microarray scanner at 100% laser power and 50–70% gain settings.

### Data analysis and mining

Three technical replicate microarray hybridisations per biological replicates were performed, so altogether we obtained data from nine arrays. Scan images were analysed using ArrayPro software, and the raw signals were within-array-normalised using the local regression (Loess) function of the software. Bad quality (empty or dirty) spots were manually removed and data between arrays were quantile-normalised in Excel [45]. Box and whisker plots [46] were created using an Excel template (http://www.vertex42.com/ExcelTemplates/box-whisker-plot.html). Quantile-normalised data were log2 transformed and statistical analysis was performed by the empirical Bayesian option of the web-tool ArrayMiner [47], which applies the method of Benjamini and Hochberg [48] to adjust the significance threshold to prevent false positive discoveries. A q-value, an adjusted p-value for multiple testing, as a significance score for each gene was returned, and we considered genes having a q-value smaller than 0.05 as significant discoveries. Genes displaying significant changes in expression were annotated into functional categories using the MapMan software [49], [50]. Potato microarray unigene sequences were analysed by BLAST against the *S. phureja* genome (http://www.potatogenome.net/index.php/Main_Page) for further annotation. Data for *Solanaceae* proteins and *Arabidopsis* genes and proteins were collected from the UniProt (http://www.uniprot.org) and the TAIR (http://www.arabidopsis.org/) databases, respectively. Identification of transcription factors and their binding sites in the promoter regions of the putative *Arabidopsis* orthologs of the assigned genes was performed by searching the AtcisDB [51] and AthaMap [52] databases. Protein-protein interactions were identified using the BioGrid (http://www.thebiogrid.org) and IntAct (http://www.ebi.ac.uk/intact/main.xhtml) databases, and were visualised with software Osprey [53]. Microarray data were submitted to ArrayExpress under accession number E-MEXP-3221.

### RNA gel blot analysis

RNAs quantified using a NanoDrop spectrophotometer were loaded in equal amounts (20 µg) into agarose gels, separated and blotted onto Hybond-N membranes as described [54]. To generate hybridisation probes, PCR amplifications were carried out using a *S. tuberosum* leaf cDNA library [55] as template and gene-specific primers (Table S6). PCR products were isolated from agarose gels using the GFX PCR DNA and Gel Band Purification Kit (GE Healthcare), and radioactively labelled by random priming [54]. Hybridisation was carried out in Church buffer [56] at 65°C for overnight. The filter was washed for 20 min at 65°C twice in 2xSSC [54] containing 0.1% (v/v) SDS and once in 0.2xSSC with 0.1% (v/v) SDS.

## Supporting Information

Figure S1 **Between array normalisation of microarray data.** Data for *TPS1*-transgenic (A) and wild-type plants (B) from nine microarrays were quantile normalised.
(PDF)

Figure S2 **Pathway map of carbohydrate metabolism.** The map is based on the KEGG database (http://www.genome.jp/kegg/pathway.html). Red, green and black letters and dots represent increased, decreased and unchanged gene expression and carbohydrate levels, respectively, in *TPS1*-transgenic potato leaves when compared with the wild-type. I, inositol; F, fructose; G, galactose; Gl, glucose; M, maltose; Mt, malate; S, sorbitol; Su, sucrose; FBA, fructose-bisphosphate aldolase; PDH, pyruvate dehydrogenase; PGK, phosphoglycerate kinase; RBC, RuBisCo; SUS, sucrose synthase.
(PDF)

Table S1 **List of** differentially expressed genes not assigned to functional categories using MapMan software.
(DOC)

Table S2 **Functional annotation of differentially expressed potato genes**. Three approaches were used, functional grouping using MapMan software, BLAST searching of the *S. phureja* genome and searching the UniProt database for *Solanaceae* orthologs.
(XLS)

Table S3 **Transcription factors binding to the promoter region of putative** ***Arabidopsis*** **orthologs of differentially expressed potato genes.**
(XLS)

Table S4 **Transcription factor binding sites in the promoter region of putative** ***Arabidopsis*** **orthologs of differentially expressed potato genes.**
(XLS)

Table S5 **Protein-protein interactions involving putative** ***Arabidopsis*** **orthologs of potato proteins encoded by assigned genes in microarray experiments.**
(XLS)

Table S6 **PCR primers used to amplify cDNA fragments to generate hybridisation probes for verification of the microarray results.**
(DOC)
