# Supplementary material for: Transcriptome Analysis of Potato Leaves Expressing the Trehalose-6-Phosphate Synthase 1 Gene of Yeast
Source: PLoS One. 2011 Aug 16;6(8):e23466. doi: 10.1371/journal.pone.0023466 (PMC3156770; doi:10.1371/journal.pone.0023466)
Supplement: Figure S1 — Between array normalisation of microarray data. Data for TPS1-transgenic (A) and wild-type plants (B) from nine microarrays were quantile normalised. (PDF) [file pone.0023466.s001.pdf]

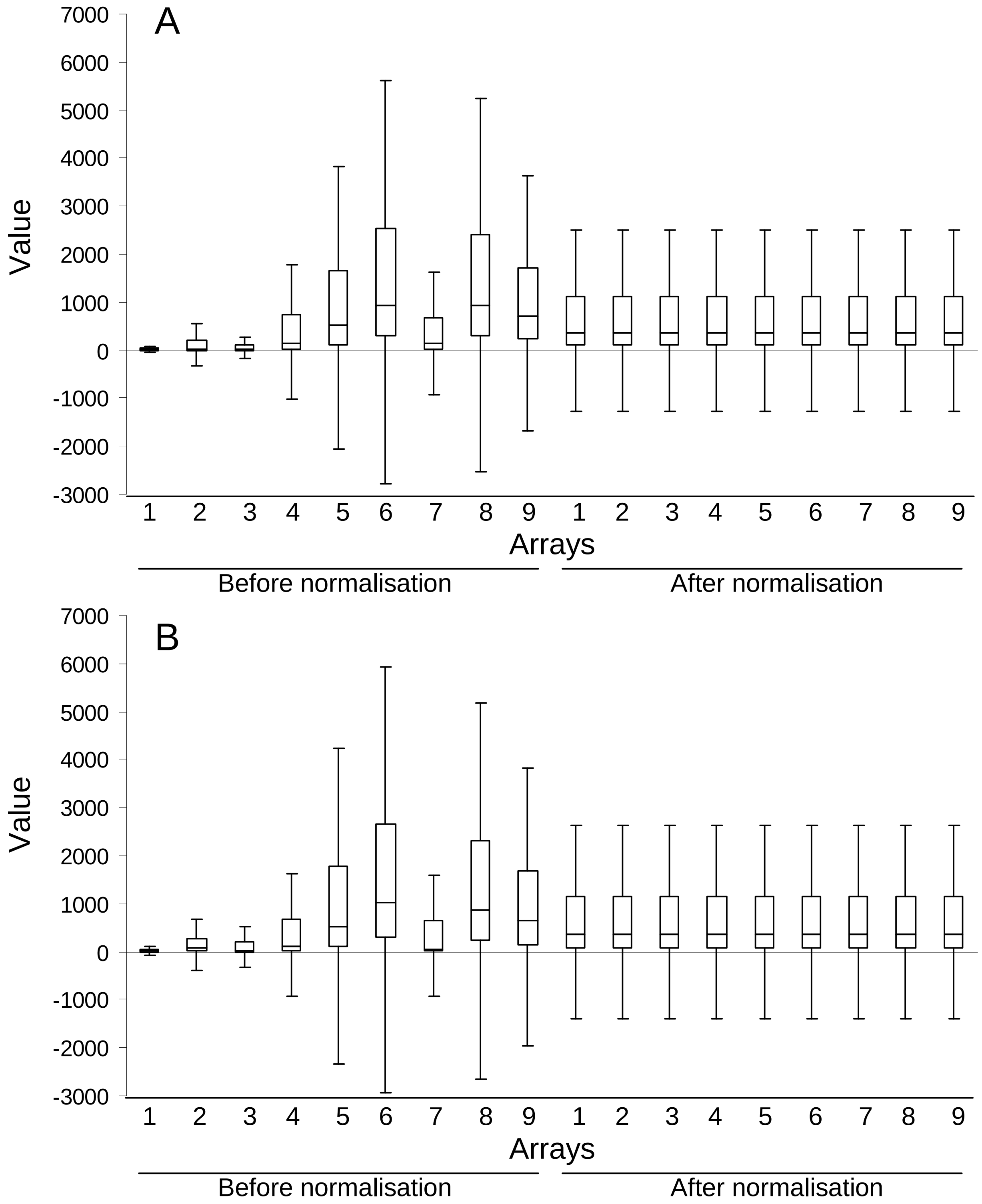

Figure S1. Between array normalisation of microarray data. Data for *TPS1*-transgenic **(A)** and wild-type plants **(B)** from nine microarrays were quantile normalised.
