# Supplementary material for: Transcriptome Analysis of Potato Leaves Expressing the Trehalose-6-Phosphate Synthase 1 Gene of Yeast
Source: PLoS One. 2011 Aug 16;6(8):e23466. doi: 10.1371/journal.pone.0023466 (PMC3156770; doi:10.1371/journal.pone.0023466)
Supplement: Figure S2 — Pathway map of carbohydrate metabolism. The map is based on the KEGG database (http://www.genome.jp/kegg/pathway.html). Red, green and black letters and dots represent increased, decreased and unchanged gene expression and carbohydrate levels, respectively, in TPS1-transgenic potato leaves when compared with the wild-type. I, inositol; F, fructose; G, galactose; Gl, glucose; M, maltose; Mt, malate; S, sorbitol; Su, sucrose; FBA, fructose-bisphosphate aldolase; PDH, pyruvate dehydrogenase; PGK, phosphoglycerate kinase; RBC, RuBisCo; SUS, sucrose synthase. (PDF) [file pone.0023466.s002.pdf]

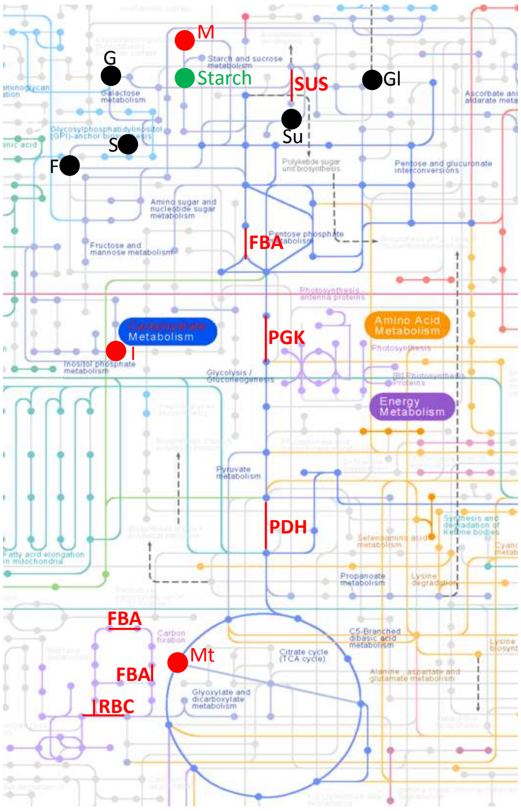

Figure S2. Pathway map of carbohydrate metabolism.
The map is based on KEGG database [http://www.genome.jp/kegg/pathway.html].
Red, green and black letters and dots represent increased, decreased and unchanged gene expression and carbohydrate levels, respectively, in *TPS1*-transgenic potato leaves compared to the wild-type.
Abbreviations: I, inositol; F, fructose; G, galactose; Gl, glucose; M, maltose; Mt, malate; S, sorbitol; Su, sucrose; FBA, fructose-bisphosphate aldolase;
PDH, pyruvate dehydrogenase; PGK, phosphoglycerate kinase; RBC, RuBisCo;
SUS, sucrose synthase.
