# Supplementary material for: Transcriptome Analysis of Potato Leaves Expressing the Trehalose-6-Phosphate Synthase 1 Gene of Yeast
Source: PLoS One. 2011 Aug 16;6(8):e23466. doi: 10.1371/journal.pone.0023466 (PMC3156770; doi:10.1371/journal.pone.0023466)
Supplement: Table S1 — List of differentially expressed genes not assigned to functional categories using MapMan software. (DOC) [file pone.0023466.s003.doc]

**Table S1. List of differentially expressed genes not assigned to functional categories by software MapMan.**

__________________________________________________________________________________________________________________

MapMan TPS/wt ratio Q-value At number Description

bin Code Log2 value __________________________________________________________________________________________________________________35.1 2.55 4.0e-02 AT3G62810 LVR family protein

35.1 2.83 4.0e-02 AT1G07080 Gamma interferon responsive lysosomal thiol

reductase family protein / GILT family protein

35.1 3.64 2.2e-02 AT2G36540 NLI interacting factor (NIF) family protein

35.1.21 -3.17 3.3e-02 AT3G46540 Epsin N-terminal homology (ENTH)

domain-containing protein/clathrin assembly protein

35.2 4.32 1.8e-02 No Hits Found Unknown

35.2 1.89 3.2e-02 No Hits Found Unknown

35.2 5.64 3.6e-02 No Hits Found Unknown

35.2 1.55 2.0e-02 No Hits Found Unknown

35.2 4.64 3.1e-02 AT3G11810 Similar to unknown protein

35.2 4.06 1.2e-02 No Hits Found Lycopersicon abscisic stress ripening protein3 (ASR3)

35.2 -5.99 9.5e-03 AT3G48180 Similar to unknown protein

35.2 3.18 1.7e-02 No Hits Found Unknown

35.2 3.83 1.4e-02 No Hits Found Unknown

35.2 3.06 4.2e-02 AT3G09140 Similar to unknown protein

35.2 2.83 4.0e-02 AT1G67785 Similar to unknown protein

35.2 2.32 4.8e-02 No Hits Found Nicotiana Africana maturase K

35.2 3.06 2.2e-02 No Hits Found Unknown

35.2 -5.65 2.2e-02 No Hits Found Unknown

35.2 3.83 1.7e-02 No Hits Found Unknown

35.2 2.47 4.0e-02 AT1G48200 Hypothetical protein

35.2 3.32 7.5e-03 No Hits Found Unknown

35.2 3.18 2.2e-02 No Hits Found Unknown

35.2 1.78 4.0e-02 No Hits Found Unknown

35.2 1.09 4.3e-02 No Hits Found Unknown

35.2 4.32 1.5e-03 No Hits Found Unknown

35.2 2.06 4.2e-02 No Hits Found Unknown

35.2 3.18 1.9e-02 No Hits Found Unknown

35.2 3.47 3.1e-02 No Hits Found Unknown

35.2 4.06 3.1e-03 No Hits Found Unknown

35.2 -4.07 2.1e-02 AT3G48180 Similar to unknown protein

35.2 2.83 3.7e-02 No Hits Found Nicotiana plumbaginifolia Guanine nucleotide-

binding protein subunit beta-like protein

35.2 -3.69 4.5e-02 AT4G17940 Binding protein

35.2 -2.40 3.7e-02 No Hits Found Unknown

35.2 -2.33 2.1e-02 AT4G37090 Similar to unknown protein

35.2 -5.33 1.5e-03 AT4G22890 Contains domain Zinc beta-ribbon

35.2 4.06 1.4e-02 No Hits Found Unknown

35.2 3.47 1.3e-02 AT3G61870 Putative protein

35.2 3.83 4.1e-03 No Hits Found Unknown

35.2 -2.09 3.1e-02 AT1G54520 Similar to unknown protein

35.2 -2.81 4.0e-02 AT2G14910 Similar to unknown protein

35.2 1.43 4.9e-02 No Hits Found Unknown

35.2 1.39 3.5e-02 No Hits Found Unknown

35.2 -3.96 9.5e-03 No Hits Found Unknown

35.2 -2.35 3.7e-02 No Hits Found Unknown

35.2 -3.31 2.0e-02 No Hits Found Unknown

35.2 3.64 2.2e-02 AT2G36540 NLI interacting factor (NIF) family protein

__________________________________________________________________________________________________________________
