# Supplementary material for: Transcriptome Analysis of Potato Leaves Expressing the Trehalose-6-Phosphate Synthase 1 Gene of Yeast
Source: PLoS One. 2011 Aug 16;6(8):e23466. doi: 10.1371/journal.pone.0023466 (PMC3156770; doi:10.1371/journal.pone.0023466)
Supplement: Table S6 — PCR primers used to amplify cDNA fragments to generate hybridisation probes for verification of the microarray results. (DOC) [file pone.0023466.s008.doc]

**Table S6. PCR primers used to amplify cDNA fragments to generate hybridisation probes for verification of the microarray results.**

| **Genes** | **Primers** |
| --- | --- |
| *rbcS* | 5’ggccaccaattaacatgaagaagt 3’  5’aaggaaaacggaaaatgccaacag 3’ |
| *SUS3* | 5’accgggcacgtaatggtgagc 3’  5’cccactgaggcacattg 3’ |
| *Snakin2* | 5’tgctccttctcgagcaagtccaat 3’  5’acgtttgttgccatgagtagtc 3’ |
| *CAT2* | 5’atacgacaccccgtttcttg 3’  5’agggcacgacttagcatcac 3’ |
| *SRG1* | 5’tccaccatgtccacaaccagaca 3’  5’tctgcggttcctccaacgcc 3’ |
| *C2C2 GATA* | 5’acggtccagacaggccggaa 3’  5’accgcaaccgagctagacct 3’ |
| *AGL8* | 5’gagtccagttgaagcgaatagag 3’  5’gtggcaaaacaaatgtagatgagt 3’ |
| *14-3-3* | 5’caagttggctgagcaggctgag 3’  5’gtctgattggatgagtaggag 3’ |
